# Selective Laser Melting Fabrication of Porous Ti6Al4V Scaffolds With Triply Periodic Minimal Surface Architectures: Structural Features, Cytocompatibility, and Osteogenesis

**DOI:** 10.3389/fbioe.2022.899531

**Published:** 2022-05-26

**Authors:** Jia Lv, Wenxuan Jin, Wenhao Liu, Xiuyu Qin, Yi Feng, Junjun Bai, Zhuangzhuang Wu, Jian Li

**Affiliations:** ^1^ Department of Orthopedics, Second Hospital of Shanxi Medical University, Taiyuan, China; ^2^ Shanxi Key Laboratory of Bone and Soft Tissue Injury Repair, Department of Orthopedics, Second Hospital of Shanxi Medical University, Taiyuan, China

**Keywords:** porous scaffolds, additive manufacturing, selective laser melting, triply periodic minimal surface, TPMS

## Abstract

The relationship between pore architecture and structure performance needs to be explored, as well as confirm the optimized porous structure. Because of the linear correlation between constant C and pore architecture, triply periodic minimal surface (TPMS) based porous structures could be a controllable model for the investigation of the optimized porous structure. In the present work, three types of TPMS porous scaffolds (S, D and G) combined with four constants (0.0, 0.2, 0.4 and 0.6) were designed, and built successfully *via* the selective laser melting (SLM) technology. The designed feature and mechanical property of porous scaffolds were investigated through mathematical method and compression test. And the manufactured samples were co-cultured with rMSCs for the compatibility study. The results indicated that the whole manufacturing procedure was good in controllability, repeatability, and accuracy. The linear correlation between the porosity of TPMS porous scaffolds and the constant C in equations was established. The different TPMS porous scaffolds possess the disparate feature in structure, mechanical property and cell compatibility. Comprehensive consideration of the structure features, mechanical property and biology performance, different TPMS structures should be applied in appropriate field. The results could guide the feasibility of apply the different TPMS architectures into the different part of orthopedic implants.

## Introduction

In recent years, porous metal scaffolds have been widely used for clinical bone defect reconstruction, and their clinical significance has been confirmed due to the effective permeability and high specific surface area (SSA) of the porous structure (Yang et al., 2014a; Yang et al., 2014b; Zhang et al., 2021). Pore architecture is a basic design feature for tridimensional scaffolds aimed at tissue regeneration, merely because it is interrelated with structural and physical variables such as mechanical strength, porosity, permeability, and specific surface area (Lin et al., 2004; Hollister, 2005; Hollister, 2009; Dias et al., 2012). Moreover, differences in pore architecture will lead to disparate porosity, permeability, and specific surface area, thereby impacting the cytocompatibility and osteogenicity of the scaffold, which has been confirmed by previous studies (Lv et al., 2015a; Lv et al., 2015b). Therefore, there is a need to explore the relationship between pore architecture and the structure performance, as well as confirm the optimized porous structure.

Triply periodic minimal surfaces (TPMS) are mathematically defined surface curvatures, infinite and periodic in the 3D Euclidean space, making them available for highly controllable and homogeneous porous structure designs (Blanquer et al., 2017; Barba et al., 2019). TPMS-based porous structures have an excellent potential in clinical application due to their high permeability and specific surface area. The linear constant in TPMS equations defines the periodical change in minimal surfaces curvature. Therefore, the constant can be defined as the offsetting value of the ratio of void volume to solid volume, which determines the volume fraction of the void space in the porous structure, and then influences the mechanical strength, porosity, permeability, and specific surface area. The character of the constant in the equations make the TPMS-based porous structure a controllable model for the investigation of the optimized porous structure.

In this study, three types of TPMS porous structures combined with four constants were manufactured by additive manufacturing technology, and the physical features, mechanical property, and histocompatibility of the twelve sets of porous TPMS structures were investigated.

## Materials and Methods

### Design and Manufacturing of the Porous Scaffolds

Three different TPMS architectures were selected in this study: Fischer–Koch S (S) geometry, Diamond (D) geometry, and Gyroid (G) geometry. The surface equations are listed in Table 1 according to the literature (Blanquer et al., 2017). For each TPMS architecture, four different constants C were considered (0.0, 0.2, 0.4, and 0.6) to produce discrepant porosity of the porous structure. Finally, 12 different geometries (three TPMS × four constants C) were investigated in this study.

**TABLE 1:** Equations for three TPMS architectures.

TPMS structures	Equations for TPMS: *f (x, y, z) = C*
Fischer–Koch S (S)	*cos(2x).sin(y).cos(z)+cos(2y).sin(z).cos(x)+cos(2z).sin(x).cos(y) = C*
Diamond (D)	*sin(x).sin(y).sin(z)+sin(x).cos(y).cos(z)+cos(x).sin(y).cos(z)+cos(x).cos(y).sin(z) = C*
Gyroid (G)	*cos(x).sin(y)+cos(y).sin(z)+cos(z).sin(x) = C*

**FIGURE 1:**
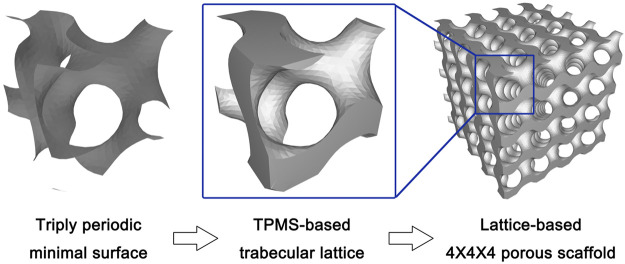
The schematic diagram of design process from triply-periodic minimal surface (TPMS) to TPMS-based porous scaffolds.

The surface equations were input into software for the generating of TPMS STL files, which were then used for the porous structure production. The surface in TPMS geometry is defined as the interface between the solid region and the pore region in the porous structure. Then, the boundary of the solid part in each TPMS was sealed to produce one single lattice, which is 2.5*2.5*2.5 mm in volume. Then, the final porous scaffold was produced through the repetition of one single lattice four times in three directions (X, Y, and Z direction), which finally contained 64 lattices with 10*10*10 mm (1000 mm^3^) in total volume (Figure 1).

After designing the 12 types of TPMS porous scaffolds, the STL files were used for the manufacturing. A selective laser melting (SLM) apparatus (EP-260, E-Plus-3D, China) equipped with a laser beam of 100 μm in diameter was used to fabricate the scaffolds. The material used in this study is Ti6Al4V powder (4.5 g/cm^3^), a metallic powder widely used in medical implants.

### Structural Features Characterization

The designed total solid volume (mm^3^) and surface area (mm^2^) of the TPMS porous scaffolds were acquired from data in the STL files. Then, the designed porosity (1—designed total solid volume/1000 mm^3^) and specific surface area (designed surface area/1000 mm^3^) were calculated. The designed permeability was then mathematical calculated using the following formula: (designed porosity)^3^/[2 * (designed specific surface area)^2^], which has been widely used as a reference (Martys et al., 1994).

The densitometric method was used to determine the actual porosity of the SLM-made specimen, and the following calculation was made: porosity = 1—(density of the porous scaffold/density of a solid scaffold with the same size). Based on the measured (Dm) and designed (Dd) porosity data, the deviation in dimension was calculated as follows: offset = [(Dd–Dm)/Dd] *100%.

Regarding the analysis of the mechanical property, the finite element analysis and the compression test were conducted. A 10 Newtons (N) vertically downward stress was loaded onto the TPMS porous scaffolds model for the finite element analysis, and the stress distribution and displacement distribution of each porous scaffold under stress loading conditions were investigated. For the finite element analysis, the materials parameters were set to be elastic modulus of 103400 MPa and Poisson ratio of 0.35. The boundary conditions were set to be that the bottom of the porous scaffold was immobilized, and a vertical force was applied on the top surface of the porous scaffold. Compression tests were performed on the 3D printed specimens (10*10*10 mm). For this purpose, an Instron 5969 machine was used. The specimens were compressed at a constant strain rate of 10^−2^ s^−1^. Three repeats were performed for each group.

### Cell Culture and Seeding

The rat bone marrow-derived mesenchymal stem cells (rMSCs, Cyagen Biosciences Inc., China) were cultured in Dulbecco’s Modified Eagle’s Medium (DMEM) supplemented with 10% FBS and 1% penicillin/streptomycin. The scaffolds (10*10*10 mm) were first soaked in the culture medium for 1 h before cell seeding. Then, the samples were placed one per well in 24-well culture plates, and a 100-μl droplet of the culture medium containing a certain number of cells was placed on the scaffold. The unattached cells were pipetted and placed on the top of the specimens again. The pipetting procedure was repeated four times at every 30 min interval. The cells were allowed to attach for 2 h in an incubator at 37°C with 5% CO_2_. Each well was then supplied with 1 ml culture medium and further cultured for 22 h to promote cell spreading on the scaffold. Then, the cell-seeded samples were supplied with 1 ml fresh growth medium and cultured at 37°C with 5% CO_2_ for different experimental periods during which refreshment was made twice a week.

### Cytocompatibility Studies

In this part of the study, 5*10^4^/100 μl rMSCs were seeded on each scaffold. After 1, 4, and 7 days of co-culture, the viability of the cells cultured in 3D TPMS porous scaffolds were assessed using the Cell Counting Kit (CCK8; Invitrogen, United States). Briefly, at various intervals, the culture wells in each group were replenished with fresh medium containing CCK-8 solution, as recommended in the manufacturer’s procedure. After one hour incubation at 37°C, the solution absorbance was measured at 450 nm.

After 24 h of culture, the cells of each group were trypsinized and pooled for cell cycle analysis. The cells were stained with propidium iodide (PI, Cell Cycle and Apoptosis Analysis Kit, Beyotime, China), and the PI-elicited fluorescence of individual cells was measured using flow cytometry (FACS Canto Ⅱ, BD). After 7 days of co-culture, the Calcein-AM/PI Double Stain Kit (Yeasen, China) was used for live/dead assay using flow cytometry (FACS Canto Ⅱ, BD).

### Osteogenic Ability

In this part of the study, 1*10^5^/100 μl rMSCs were seeded on each scaffold. After 4 days of co-culturing for cell proliferation, the osteogenic medium (Cyagen Biosciences Inc., China) was used to induce osteogenic differentiation of rMSCs. Briefly, cell-seeded scaffolds were cultured in DMEM supplemented with 10% FBS, 1% penicillin/streptomycin, 2% L-glutamine, 0.5% ascorbate, 0.5% dexamethasone, and 1% b-glycerophosphate. The culture medium was changed twice a week.

The influence of the scaffolds on osteogenic differentiation of the rMSCs was assessed by evaluating the ALP activity and calcium deposition of the extracellular matrix (ECM). The former was carried out after 10 days of osteogenic induction culture using an Alkaline Phosphatase Assay Kit (Beyotime, China). The cells on the scaffolds were washed twice with PBS and lysed according to the manufacturer’s procedure. The ALP in the lysate reacted with the substrate p-nitrophenyl phosphate (pNPP) and produced colorimetric p-nitrophenol products, which were measured at 405 nm on a microplate reader. The cellular ALP activity was then normalized to the total protein concentration of cells determined by a MicroBCA protein assay kit (Beyotime, China). Regarding the ECM calcium deposition assay, the scaffolds after 14 days of osteogenic induction culture were incubated in 1 ml 0.5 N hydrochloric acid overnight to extract calcium. The total calcium content in the suspension was determined using a Calcium Colorimetric Assay Kit (Beyotime, China) according to the manufacturer’s guidelines, and was then normalized to the total protein concentration of cells.

### Statistical Analysis

The results were reported as the mean ± SD. Statistical analysis was performed using the one-way ANOVA. *p* < 0.05 was considered statistically significant.

## Results

### TPMS Scaffolds Designs and the Geometrical-Physical Parameters

**FIGURE 2:**
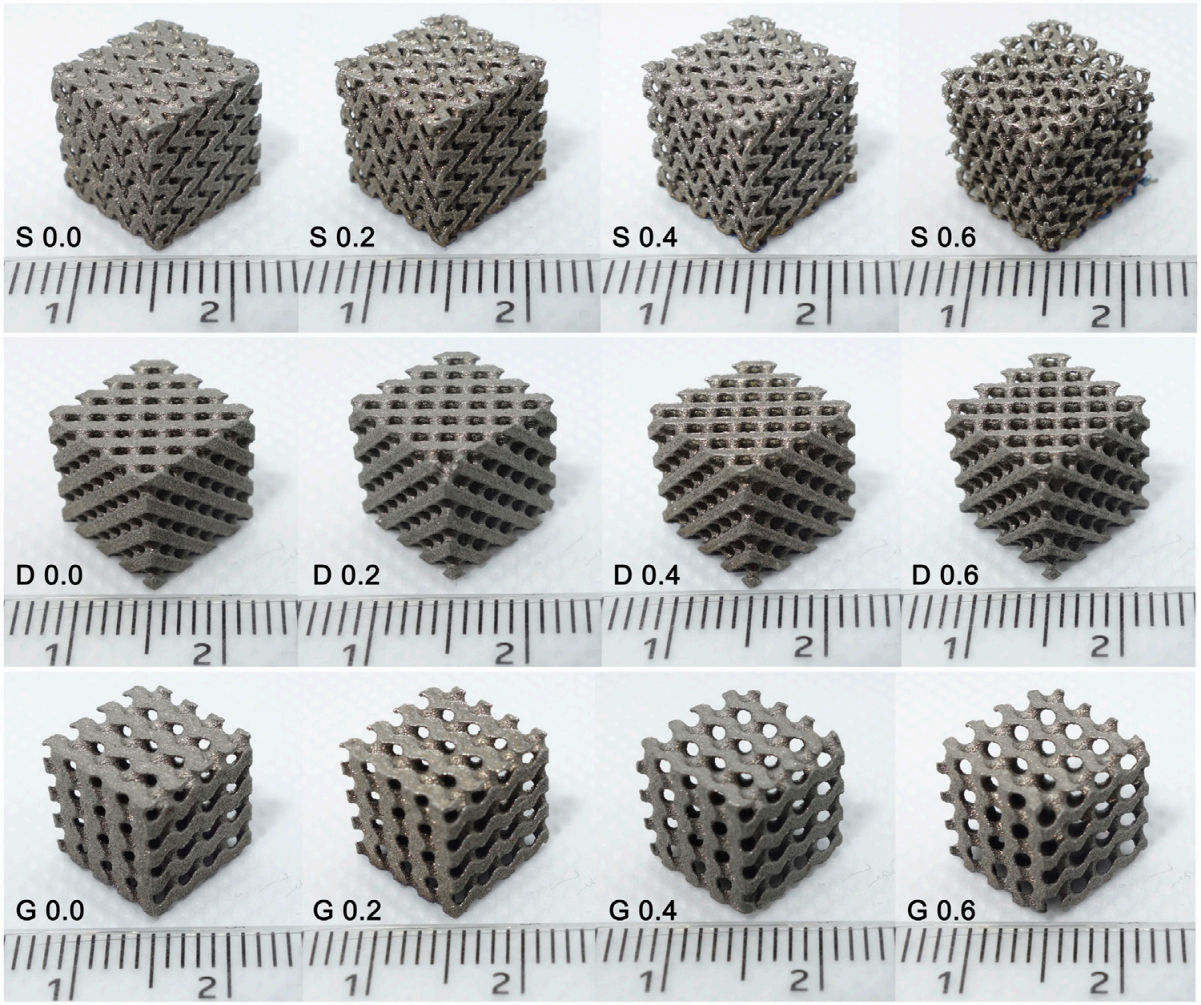
Samples manufactured by selective laser melting. The appearance of the manufactured specimens was basically consistent with the designed models that were presented in Figure 3.

**FIGURE 3:**
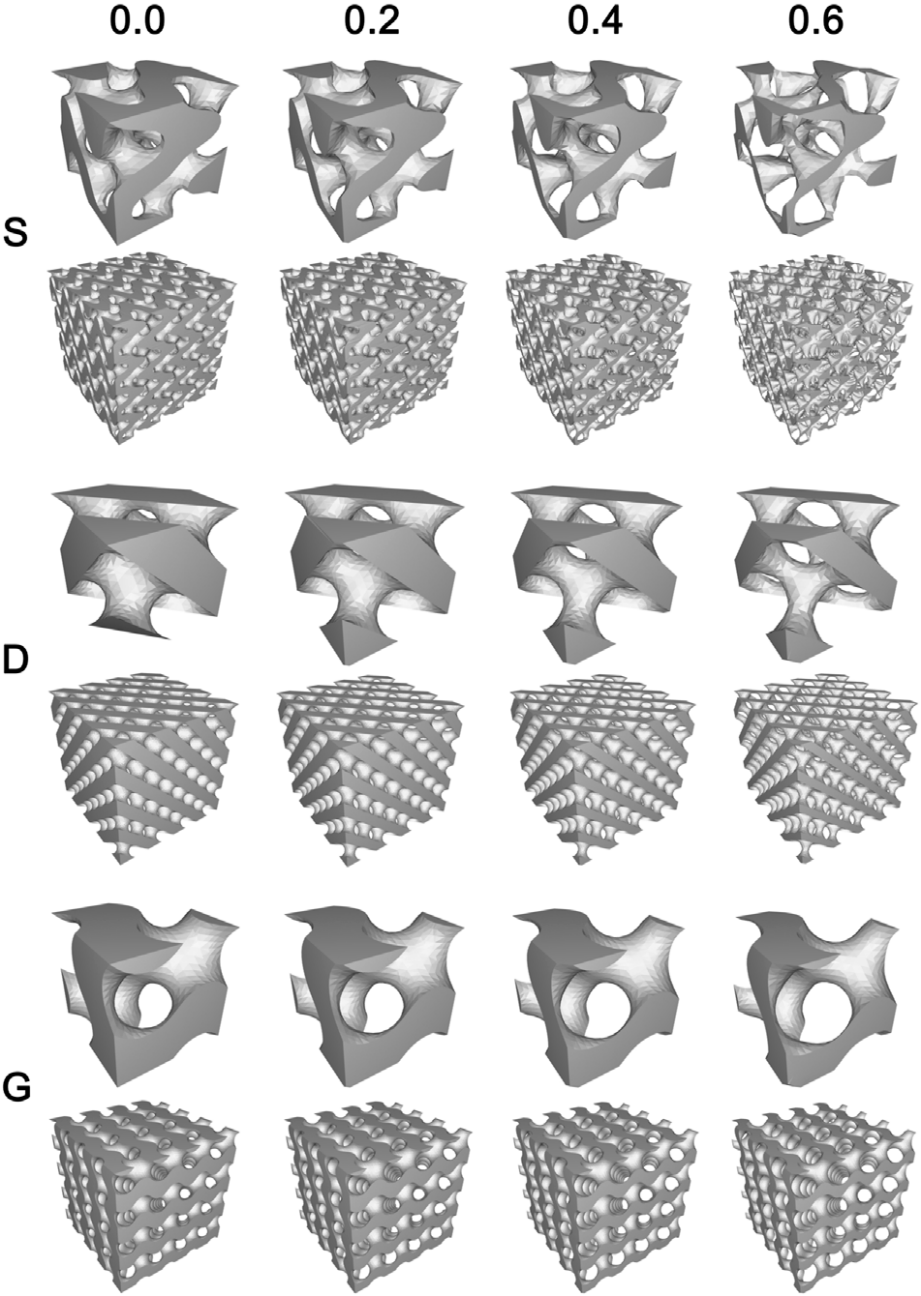
The final design diagram (stl. models) of twelve samples (three architectures combined four constants). The gradient variation of porous structures was clearly presented.

Three types of TPMS porous structures combined with four constants were designed and subsequently manufactured using the SLM technology. The results showed that the appearance of the manufactured samples (Figure 2) was basically consistent with the designed models (Figure 3). Specifically, the designs features that both the pore structure appearance and the gradient changes of the pore dimension between different constants, were all clearly reflected in the final made samples.

In the porous scaffolds, TPMS form a void/solid interface where the void space represents the pore channel of the porous scaffold, which is for cell adhesion and bone ingrowth, and the solid space represents the bone trabecula that is materialized by the Ti6Al4V powder. The different TPMS interface morphology leads to the differences in pore channel geometry in porous scaffold, with a more tortuous channel geometry in the S porous structure, and a more straightly open channel geometry in the D and G porous structures.

**FIGURE 4:**
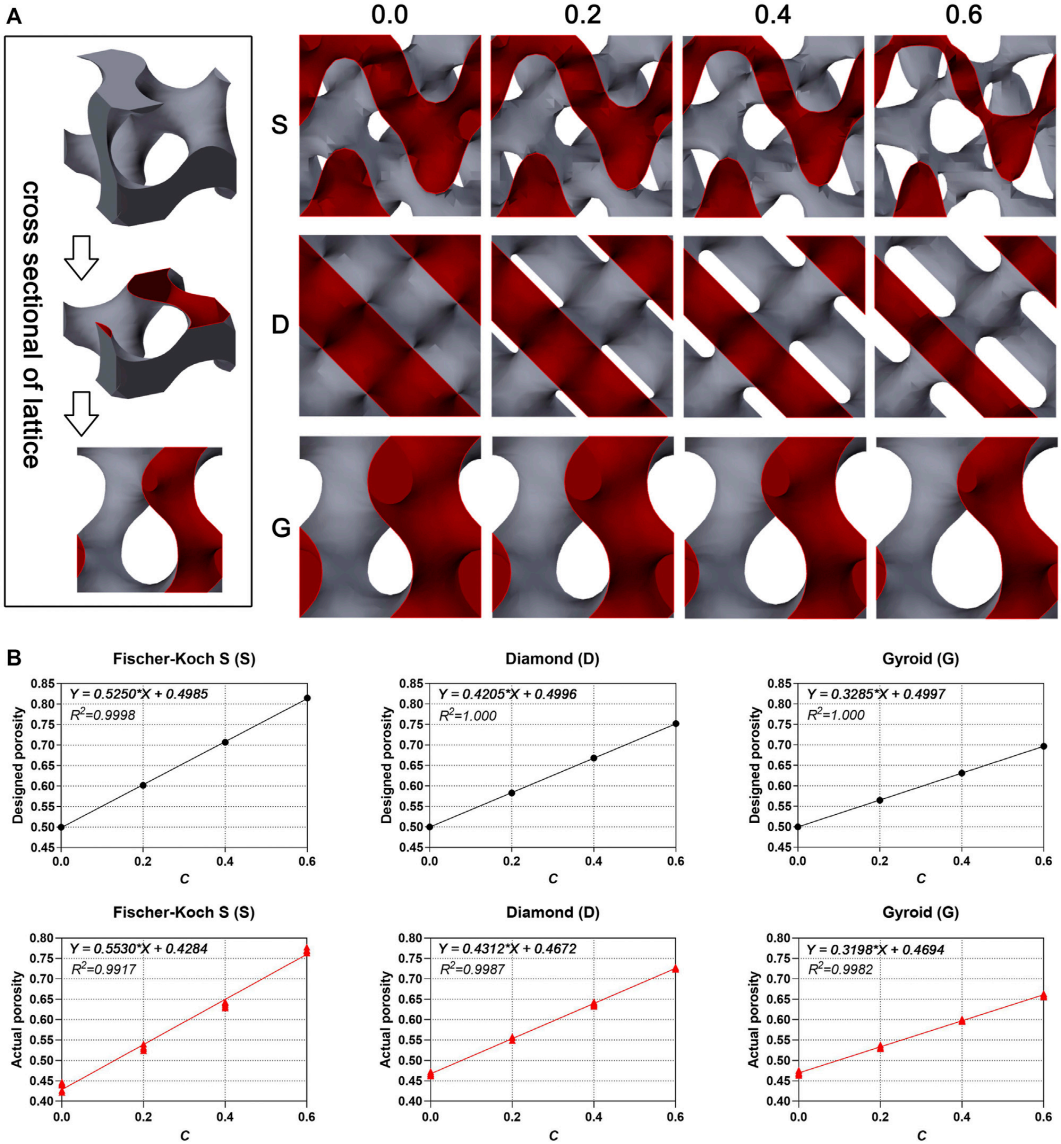
**(A)** The cross-sectional diagram of single lattice. **(B)** The linear correlation between designed porosity, actual porosity (*n* = 6) and constant C.

The results indicate that the porosity increased by the value of constant C for all type of porous scaffolds (Figure 4 and Table 2). In this study, the single lattice model diagram was sliced in half from the middle for the visual display of the porosity variation (Figure 4A). In the cross-sectional drawing, the red part represents the solid space, and remaining part represents the void space. The results showed that the ratio of the void part was equal to the ratio of the solid part, as the constant C is 0.0 (0.5 of porosity), and the ratio of the solid part decreased as the constant C increased (Figure 4B). The actual porosity of the manufactured porous scaffolds was also calculated (Figure 4B and Table 2). The results indicated that the actual porosity of all samples was slightly lower than the designed porosity. In all type of samples, the deviation decreased as the constant C increased. Furthermore, S surface-based porous scaffolds have a higher deviation in actual porosity than the other two surfaces in every group of constant C, indicating the more difficult precision manufacturing of the S surface. It needs to be emphasized that, both the designed porosity and the actual porosity were linearly correlated with the constant C (R^2^ value almost as and even equal to one). It indicates the feasibility in controlling the porosity by adjusting the constant in the linear equation of porosity.

**TABLE 2:** Designed porosity, actual porosity (*n* = 6) and deviation (*n* = 6, %).

*C* Value	S			D			G		
	Designed	Actual	Deviation	Designed	Actual	Deviation	Designed	Actual	Deviation
0.0	0.500	0.437 ± 0.010	12.60 ± 2.02	0.500	0.466 ± 0.004	6.74 ± 0.86	0.500	0.470 ± 0.004	6.07 ± 0.78
0.2	0.602	0.532 ± 0.005	11.65 ± 0.87	0.583	0.555 ± 0.003	4.84 ± 0.56	0.565	0.533 ± 0.003	5.77 ± 0.54
0.4	0.707	0.638 ± 0.006	9.73 ± 0.85	0.668	0.639 ± 0.004	4.34 ± 0.62	0.631	0.598 ± 0.002	5.26 ± 0.39
0.6	0.815	0.770 ± 0.004	5.48 ± 0.54	0.752	0.726 ± 0.002	3.51 ± 0.24	0.697	0.661 ± 0.004	5.16 ± 0.56

**FIGURE 5:**
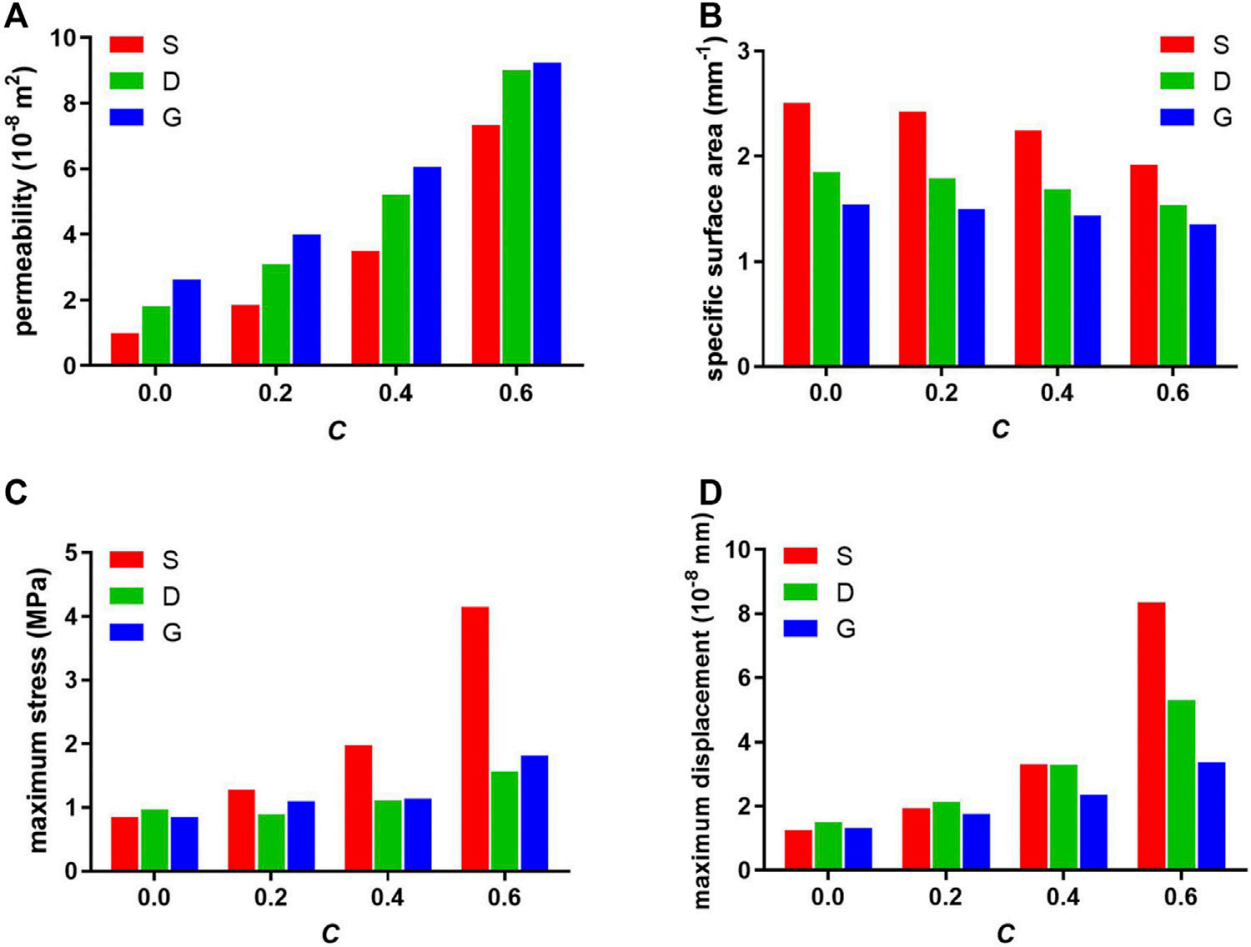
Designed **(A)** permeability, **(B)** specific surface area, and finite element analyzed **(C)** maximum stress, **(D)** maximum displacement of porous scaffolds.

The constant C determines the ratio of void volume to solid volume, and therefore determines the porosity of the porous structure and influences the permeability and specific surface area. As shown in Figure 5A and Figure 5B, as the constant C increased, the permeability increased and the SSA decreased in each structure. Furthermore, among the three types of porous scaffolds, the S surface-based porous scaffold possesses the largest SSA and the least permeability in each group of constant C, contrary to the G surface-based porous scaffold. Moreover, the SSA value of S scaffolds at constant C 0.6 was even higher than that of D and G scaffolds at constant C 0.0.

### Mechanical Property of Scaffolds

**FIGURE 6:**
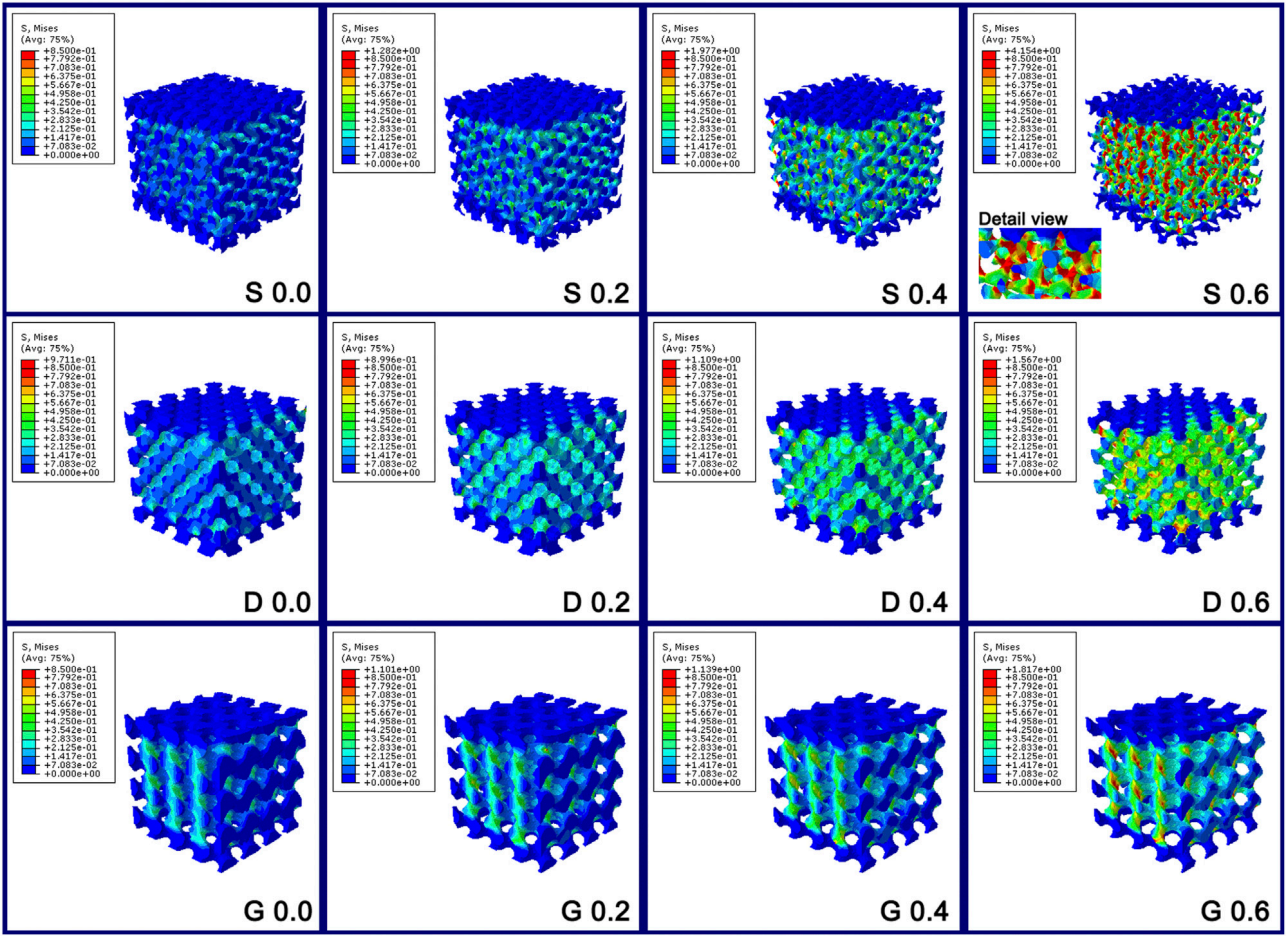
The stress distribution diagram of the porous scaffolds through the finite element analysis. The stress variation tendency in S porous scaffolds was steep; however, that of D and G porous scaffolds were smooth.

The mechanical property of porous scaffolds was studied through the finite element analysis. Figure 5C and Figure 6 show the stress distribution of the porous scaffolds. In this study, the blue part is defined as the low stress zone (<0.2833 MPa), the green and yellow parts as the middle stress zone (0.2833–0.6375 MPa), and the red part as the high stress zone (stress concentration, > 0.6375 MPa). The stress pattern could be explained as follow:

1) As the constant C was 0.0, the stress shielding was obvious. The loaded stress hardly conducts into the internal part of the porous structure, such that the internal structure comprised mostly of the low stress zone in three porous scaffolds. Nonetheless, compared with S porous scaffolds, a larger green zone was found in the D and G porous scaffolds.
2) As the constant C increased (0.2 and 0.4), the stress shielding was reduced, such that more middle stress zones arose in the internal structure. It should be noted that the red zone emerged obviously in S porous scaffolds when the constant was 0.4.
3) As the constant C was 0.6, the stress concentration zone increased markedly in the S porous scaffolds, which had the largest stress concentration zone and the maximum stress value. In particular, the stress concentration zone was mainly located at the maximum surface curvature region (the intersection of different struts, “Detail view” in Figure 6 “S 0.6”). Nonetheless, the red part was only slightly increased in D and G porous scaffolds.

Briefly, between the different values of constant C, the stress variation tendency in S porous scaffolds was steep; however, that of D and G porous scaffolds were smooth. The maximum stress value of S at constant C 0.4 was even higher than that of D and G at constant C 0.6 (Figure 5C).

**FIGURE 7:**
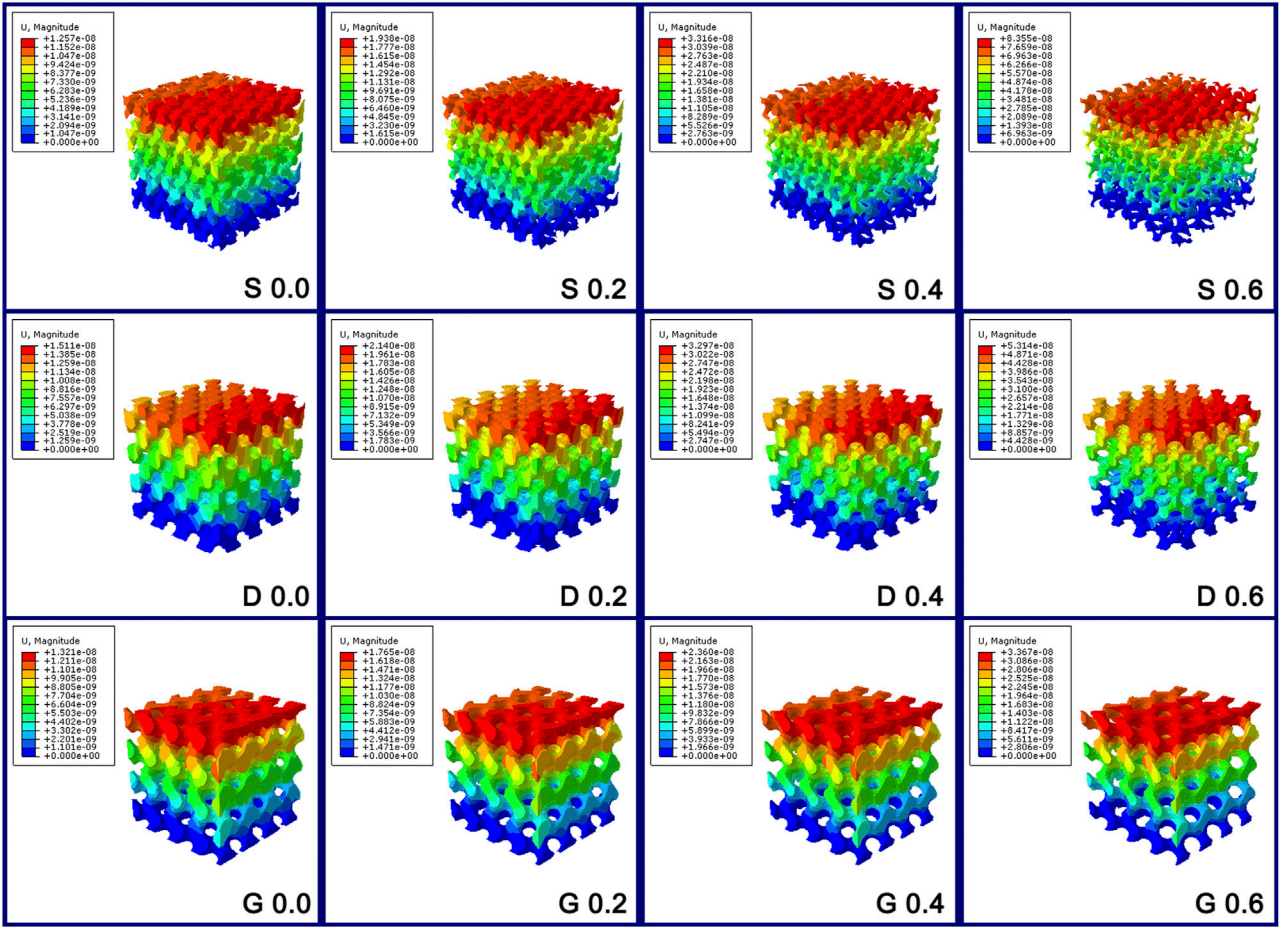
The displacement distribution diagram of the porous scaffolds through the finite element analysis. The maximum displacement value variation tendency in S porous scaffolds was steep; however, that of G porous scaffolds was smooth.

Figure 5D and Figure 7 show the displacement distribution of the porous scaffolds under the stress, which represent the stability after implantation. The maximum displacement value and displacement span (span from zero to the maximum displacement value in Figure 5D) increased as the porosity increased, indicating that the porous structures become more unstable as the solid part decreased. Similar to the results of stress distribution, between different values of the constant C, the maximum displacement value variation tendency in S porous scaffolds was steep; however, that of G porous scaffolds was smooth. The maximum displacement value and displacement span of G at constant C 0.6 was equal to that of S and D at constant C 0.4, indicating the better stability of G porous scaffolds under stress loading.

**FIGURE 8:**
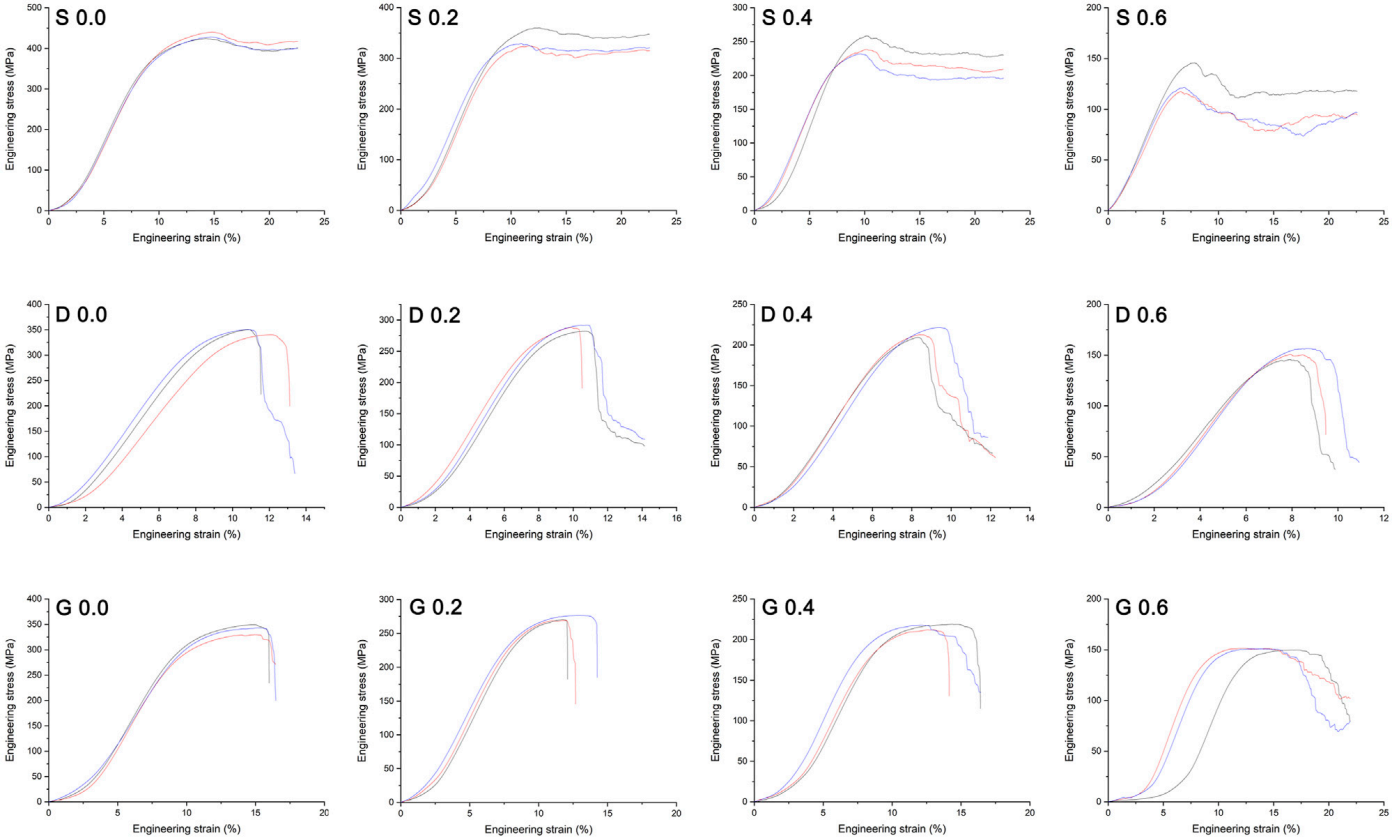
Mechanical stress–strain curves of the manufactured specimens. The peak stress at the end of initial elastic loading stage in curve is generally considered to be the bearable maximum stress of the porous scaffold before structure yielding. Three repeats were performed for each group.

Figure 8 shows the compression test results of the different manufactured specimens in the form of stress-strain curves. The stages of deformation observed could be classified as follow: 1) an initial elastic loading stage; 2) followed by a steeply declined stress region due to the yielding of the structure; 3) finally a plateau stress region followed by a slightly stress increase (especially obvious in S porous scaffolds), that due to the densification of the crushed porous structure. The peak stress at the end of initial elastic loading stage in stress–strain curve is generally considered to be the bearable maximum stress of the porous scaffold before structure yielding. For all the architectures, the peak stress decreased as the porosity increased, indicating the reduced load bearing capacity as the solid fraction decreased. Statistical values for elastic modulus measured experimentally of the different porous scaffolds are also presented in Table 3. The results of compressive test were consistent with the finite element analysis, such that the decline tendency of peak stress and elastic modulus in S porous scaffolds were steep, that of D and G porous scaffolds were smooth.

**TABLE 3:** Elastic modulus measured experimentally of the different porous scaffolds (*n* = 3, MPa).

*C* value	S	D	G
0.0	5803 ± 68	4954 ± 264	4562 ± 273
0.2	5218 ± 59	4602 ± 28	4172 ± 98
0.4	4168 ± 263	3797 ± 61	3553 ± 137
0.6	2570 ± 125	2968 ± 118	2849 ± 160

Based on the mechanical property results, the available range of constant C in S porous scaffolds is within a small extent, which tends to be from 0.2 to 0.4. However, the available range of constant C in D and G porous scaffolds is from 0.2 to 0.6, which is within a wide extent. It needs to be emphasized that, between the different constant C, the mechanical performance of G porous scaffolds under the stress loading was steady, that the variation tendency was relatively smooth in term of the finite element analysis and compression test.

### Cytocompatibility of Scaffolds

**FIGURE 9:**
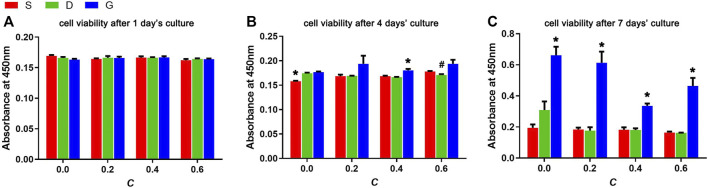
Cell viabilities of rMSCs after **(A)** 1 day’s culture, **(B)** 4 days’ culture and **(C)** 7 days’ culture on scaffolds with varied TPMS architectures (n = 3, *indicates significant differences compared with the other two groups, #indicates significant differences compared with the G group, *p* < 0.05).

The rMSCs were seeded on the scaffolds and cultured for 1, 4, and 7 days for cell viability assessment (Figure 9). In the early period (1 day), no difference was observed among the samples, which indicates the availability and consistency of the cell seeding process. At day 4, a slight but non-significant difference was observed, indicating that the G porous scaffolds show a slight advantage. A significant difference was observed after 7 days of culturing. First, the G porous scaffolds exhibited significantly higher cell viabilities than the other two TPMS in each group of constant C. In addition, compared with the other three constant C, the three types of scaffolds had the best cell viability when the constant C was 0.0.

**FIGURE 10:**
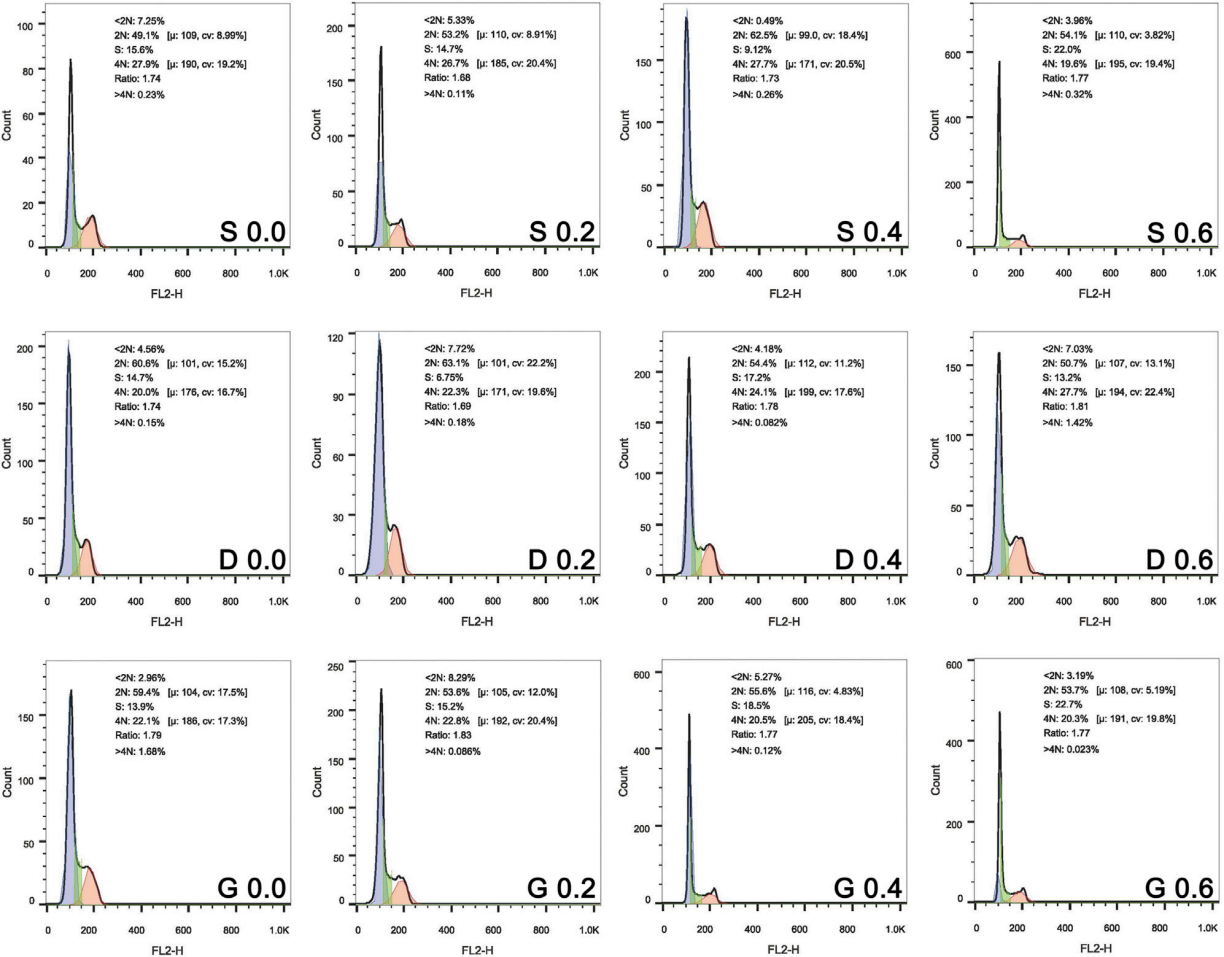
Cell cycle of rMSCs on scaffolds with varied TPMS architectures after 1 day of co-culture.

**FIGURE 11:**
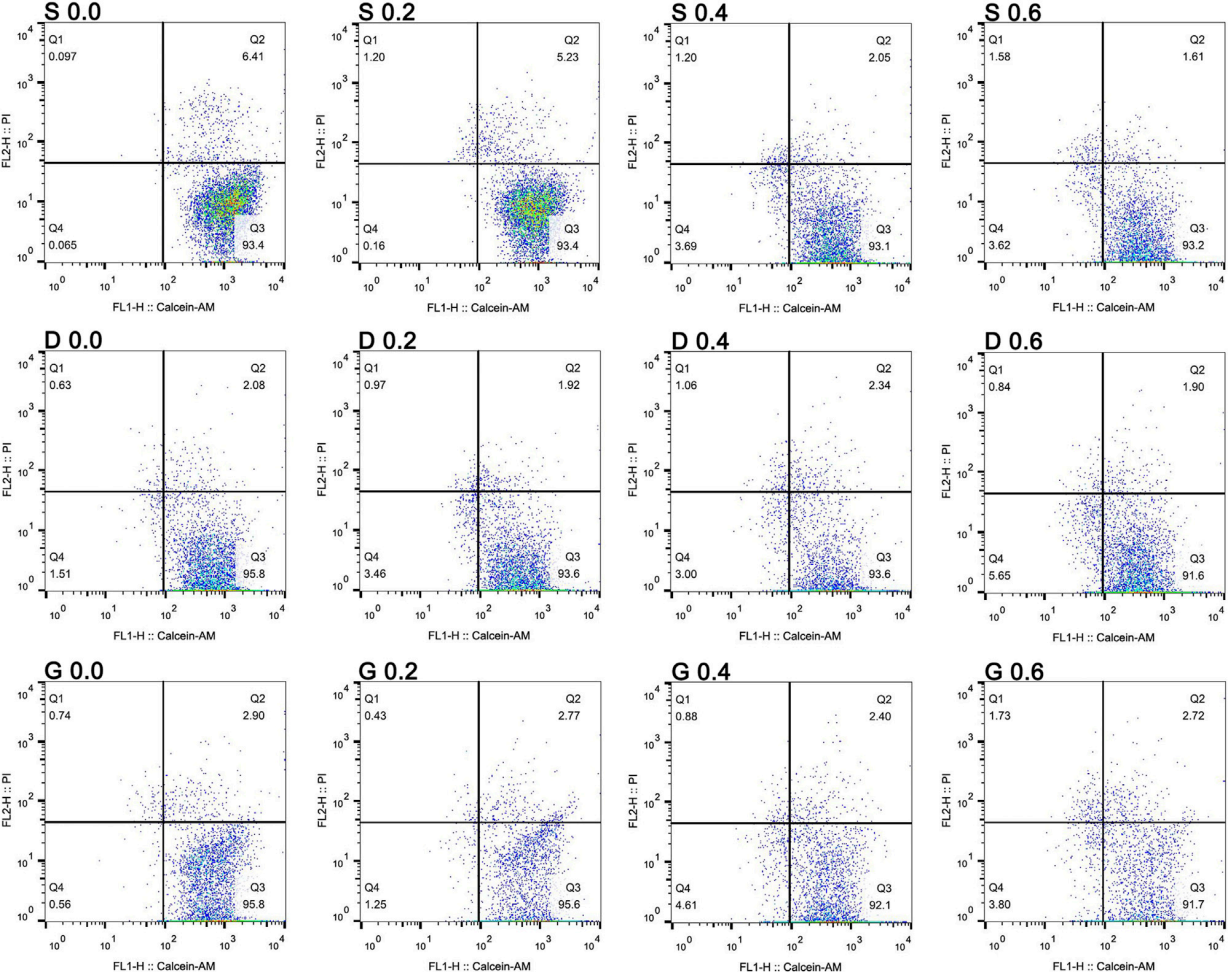
The live/dead assay of rMSCs on scaffolds with varied TPMS architectures after 7 days of co-colture.

For the further verification of the CCK results, the cell cycle after 1 day of culture and the live/dead assay after 7 days of culture was assessed (Figure 10 and Figure 11). In Figure 10, the 2N represents the G0/G1 phase of the cell cycle, 4N represents the G2/M phase of the cell cycle. Specifically, the G1 is the first gap of interphase in the cell cycle, the G2 is the second gap of interphase in the cell cycle, then the S is the synthesis phase of DNA (DNA replication) that between the G1 and G2. The M is the mitosis and cytokinesis phase of the cell cycle. The G0 represent the cells that do not divide normally, but divide when stimulus. The combined S and G2/M (S+4N) value reflects the proportion of cells in DNA synthesis and following mitosis state. In Figure 11, the Q3 value (Calcein positive cells) represents the live cells, and the Q1 value (PI positive cells) represents the dead cells. The acquired results were basically in accord with the cell viability rsults, indicating that the TPMS porous scaffolds did not have a suppressive effect on rMSCs proliferation.

### Stimulation of Osteogenic Differentiation

**FIGURE 12:**
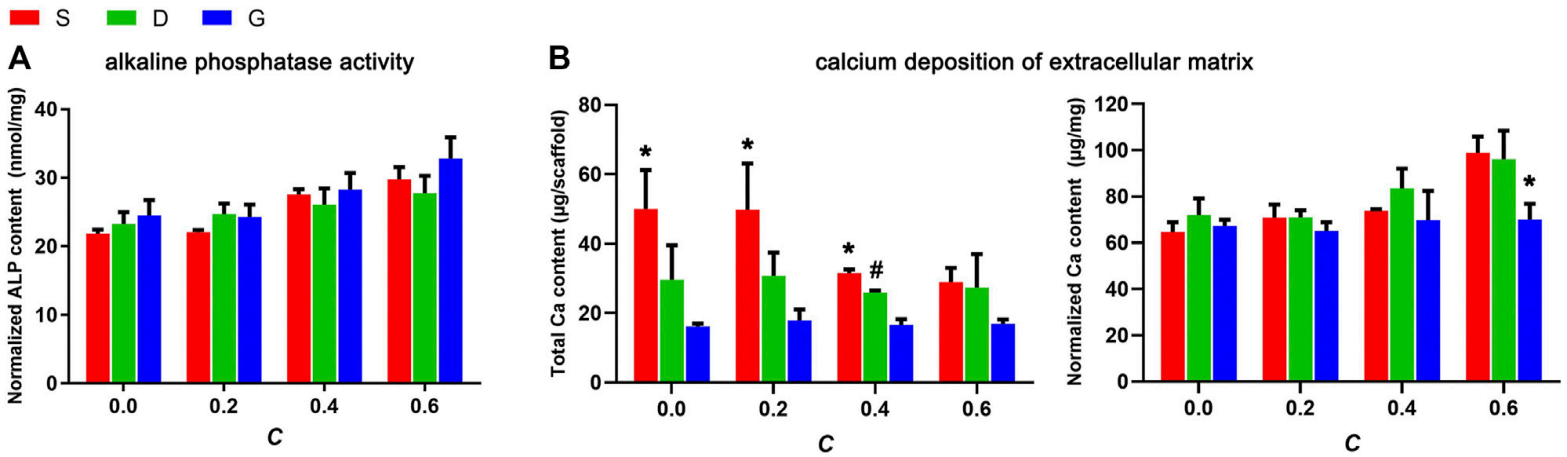
**(A)** The ALP activity of rMSCs on scaffolds after 10 days of osteogenic induction culture and, **(B)** The ECM mineralization of rMSCs on scaffolds after 14 days of osteogenic induction culture (*n* = 3, * indicates significant differences compared with the other two groups, # indicates significant differences compared with the G group, *p* < 0.05).

To elucidate the efficiency of the materials in stimulating the osteogenic differentiation of rMSCs, ALP activity and the degree of ECM mineralization was inspected (Figure 12). The results indicated that ALP activity increased as the constant C increased. In addition, the G porous scaffolds exhibited slightly higher ALP activity than the other two TPMS in each group of constant C, but no significant differences were observed.

ECM mineralization was evaluated by quantification of the calcium content of the ECM. As shown in Figure 12B, the variation tendency of the total calcium content per scaffold, either between different structures or between four constants, was basically in accord with the SSA value, indicating increased calcium deposition in a higher surface area. However, as normalized by the total protein content, an opposite tendency was observed, such that the calcium content slightly increased as the constant C increased, which is partially in accord with the permeability value. It should be noted that, for both the total calcium content or the normalized calcium content, no significant variation was observed between the different constant C in G porous scaffolds, indicating that the ECM mineralization of cells seeded on G porous scaffolds was almost not affected by the structure features.

## Discussion

To advance the field of bone defect repair, some key questions are amongst regenerative medicine researchers: firstly, the scaffold with precise and optimized properties is needed to match mechanical requirements and induce effective tissue regeneration; secondly, the metallic bone substitute scaffold that can be constructed efficiently is needed. The approach combining TPMS-based scaffold designs that embody trabecular bone-mimicking topography, and additive manufacturing tech that allows the efficient building of scaffolds with specific geometry, has become a promising solution for the achievement of the above goals (Chen et al., 2020). There have already been many reports regarding how to manufacture different TPMS scaffolds with different structure properties (Yuan et al., 2019; Chen et al., 2020), the investigation of the effect of different TPMS architectures on cell behavior is few. In this research, not only the relationship between structure features of three widely applied TPMS-based porous scaffolds and structural equation was comprehensive evaluated, but also that how different parameters of the structure can affect cytocompatibility (cell viability, osteogenic induction property) was preliminary studied.

The numerous traditional pore structures for the application in tissue engineering have been studied in past few decades. Due to the lack of a specific mechanism of pore structure affecting structure performance, the tailoring of the scaffolds to an optimized design was still not feasible. However, compared with the traditional porous architecture, the feature of constructing pore architecture by mathematical formula makes the TPMS-based porous scaffold a controllable and advantageous model for the porous structure optimization (Ambu and Morabito, 2019; Dong and Zhao, 2021). In this study, the different equations given in Table 1 require the use of a constant C, which could determine the void/solid ratio of the scaffold. Changing this constant will have different effects on each structure. More precisely, this constant defines the proportion of the solid skeleton (reflected in the porosity). Changing the constant leads to differences in porosity of the porous structure, thus impacting the permeability, SSA, and mechanical property of each structure, as revealed by the results in this study. Considering the linear correlation between porosity and constant C, which was established by our results, the feasibility of TPMS-based pore structure design optimization through the adjustment of the constant is worthy to be further studied.

Relationships between structure features and structure performance have been described in many literatures (Melchels et al., 2010; Van Bael et al., 2012). In this study, the discrepancy in pore channel architecture also accounts for the different character of each structure. According to the literature reports, the S, D, and G TPMS could be classified in two groups (Blanquer et al., 2017): S with substantially higher maximum surface curvatures and wide curvature distributions, and D and G with relatively low surface curvatures and narrow curvature distributions. Due to these specialties, the S structure possesses a more tortuous channel geometry, whereas the D and G structures possess a more straightly open channel geometry, which has been revealed in our design models and manufactured samples. The structure feature of S porous scaffolds results in a lower permeability but larger SSA, and makes it easier for them to generate the stress concentration zone and displacement at the maximum surface curvature region. On the contrary, a higher permeability, lower SSA, and more mechanically stable property were found for G scaffolds with an open pore channel architecture. In addition, in this study, the mechanical properties in each structure were basically in accord with the porosity changes. The higher porosity represents the smaller metal skeleton portion, which also explains the mechanical instability characteristics of the S structure (larger stress concentration zone, higher displacement value and lower peak stress) while the constant C was 0.6. This study also shown that a lower porosity may not mean a lower permeability if the geometry is altered, such as the difference between G scaffold and S scaffold, that is basically consistent with literature reports (Pires et al., 2021). It further indicated that the G scaffold could have the better permeability while have the more metal skeleton portion (more stable). The literature has reported that the bone remodeling process is largely dependent on the ability of bone tissues to sense and adapt to the mechanical loading (Zhang et al., 2017). Our results also proved that, while C was 0.6, the G scaffold was mechanically strong enough to load bearing, yet not too strong to have the stress shielding effect. In summary, the pore architecture based on the G TPMS can make the porous scaffold possess a good feature in permeability and mechanical performance.

One of the advanced additive manufacturing methods, the powder bed fusion technique, has great potential for metal implant fabrication, and can be used to manufacture high-quality metallic porous structure without constraints of geometry (Van der Stok et al., 2013; Wauthle et al., 2015). As the sub-class of the powder bed fusion method, SLM has been employed to build up metallic TPMS structures in biomaterial studies (Yuan et al., 2019). In this study, the samples were built successfully *via* the SLM technology in a layer-upon-layer manner. The whole manufacturing procedure was good in controllability, repeatability, and accuracy, which was confirmed by the low standard deviation of the actual porosity. However, the production of S porous scaffolds was more inaccurate, revealed by the higher deviation in actual porosity than the other two surfaces in every group of constant C. This could be explained by the more complex channel geometry in the S porous structure, which makes it challenging to remove the residual incompletely melted metal powders residing in the internal pore.

Scaffolds with higher permeability, i.e., higher mass transport potency, allow sufficient nutrient infiltration to sustain cell viability and have been shown to improve tissue regeneration (Bohner et al., 2011; Jeong et al., 2011; Mitsak et al., 2011). The minimum permeability value required for vascularization and mineralization was suggested to be 3*10^−11^ m^2^ (Hui et al., 1996; Jones et al., 2009). Due to the positive correlation with the protein adsorption, SSA remains an important factor of scaffold design in the investigation of the biological performance of porous implants (Frith et al., 2012; Lam and Segura, 2013). Moreover, the permeability was inversely related to the specific surface area of the scaffolds, which has been confirmed by our previous results (Lv et al., 2015a). In this study, the complete pore interconnectivity of the TPMS porous scaffolds contributed to the relatively high permeability in the order of 10^−9^–10^−8^ m^2^, providing an environment through which nutrients and metabolic wastes can diffuse. Moreover, the minimal surfaces property also makes the TPMS samples to have a relatively higher SSA. Our results revealed that each porous scaffold could support the proliferation and osteogenic differentiation of rMSCs; however, a few discrepancies still exist. The elevated ALP activity and elevated normalized Ca contents accompanied by the increased interconnectivity confirmed the necessity of a high permeability for tissue regeneration. However, the progressive improvement in the osteogenic performance of the S structure at a higher constant highlighted the importance of the surface area at the optimum permeability condition. Interestingly, the G porous scaffolds had more cytocompatibility with the cells, and the osteogenic performance was homogeneous among different constants. It can be hypothesized that a balance has been acquired between permeability and SSA in the G structure, and the specific mechanism needs to be further investigated.

In summary, the fabrication of porous scaffolds based on TPMS architectures with specific and controlled surface curvatures offers exciting new functionalities in the field of tissue engineering. Comprehensive consideration of the structure features, mechanical property, and biocompatibility, different TPMS structures should be applied in the appropriate field. The mechanically unstable property and the high surface area available for Ca deposition make it more appropriate to apply the S structure in the inner part of orthopedic implants. In addition, the mechanically stable property and the homogeneous osteogenic performance give the G structure a wide range of applications, which could be more appropriately applied in the load-bearing structure of the periphery part of implants. In future studies, the real tests of structural features, such that the permeability and SSA, and the expression of osteogenesis related gene, are worthy to be analyzed for the further verification of our results.

## Conclusion

A series of 12 scaffolds (three architectures with four constants) with mathematically defined TPMS architectures were designed, and were produced accurately by SLM using the biocompatible material Ti6Al4V. The linear correlation between the porosity of TPMS porous scaffolds and the constant C in equations was established. Through this new approach, the porosity of TPMS porous scaffolds can be expected to be precisely defined, which allows tailoring of the scaffolds to an optimized mechanical design. In addition to the porosity, the different TPMS architectures exhibit distinct permeability and specific surface area, which comprehensively influence cell behavior and differentiation. Finally, the results highlighted the feasibility of apply the different TPMS architectures into the different part of orthopedic implants, which mainly depend on their structure features, mechanical property, and biology performance.

## Data Availability

The original contributions presented in the study are included in the article/Supplementary Material, further inquiries can be directed to the corresponding author.
